# COVID-19 Vaccine Hesitancy and Determinants of Acceptance among Healthcare Workers, Academics and Tertiary Students in Nigeria

**DOI:** 10.3390/vaccines10040626

**Published:** 2022-04-15

**Authors:** Emmanuel O. Njoga, Philip P. Mshelbwala, Kenneth O. Abah, Olajoju J. Awoyomi, Kinley Wangdi, Shedrach B. Pewan, Felix A. Oyeleye, Haruna B. Galadima, Salisu A. Alhassan, Chinwe E. Okoli, Elisha Z. Kwaja, Onyinye S. Onwumere-Idolor, Everest O. Atadiose, Priscilla O. Awoyomi, Musawa A. Ibrahim, Kabiru M. Lawan, Shehu A. Zailani, Mohammed D. Salihu, Charles E. Rupprecht

**Affiliations:** 1 Department of Veterinary Public Health and Preventive Medicine, Faculty of Veterinary Medicine, University of Nigeria, Nsukka 410001, Nigeria; felix.oyeleye@unn.edu.ng (F.A.O.); everest.atadiose@gmail.com (E.O.A.); 2 School of Veterinary Science, University of Queensland, Gatton 4343, Australia; 3 Faculty of Veterinary Medicine, University of Abuja, Abuja 900109, Nigeria; 4 Department of Reproduction and Clinic of Farm Animals, Wrocław University of Environmental and Life Sciences, 55 Grunwaldzka St., 50-357 Wroclaw, Poland; 5 Department of Veterinary Public Health and Preventive Medicine, College of Veterinary Medicine, Federal University of Agriculture, Abeokuta PMB 2240, Nigeria; jojuawoyomi@yahoo.com; 6 Department of Global Health, National Centre for Epidemiology and Population Health, College of Health and Medicine, Australian National University, Acton Campus, Canberra 2601, Australia; kinley.wangdi@anu.edu.au; 7 College of Public Health, Medical and Veterinary Sciences, James Cook University, Townsville 4811, Australia; shedrach.pewan@my.jcu.edu.au; 8 Department of Veterinary Medicine, Faculty of Veterinary Medicine, University of Maiduguri, Maiduguri PMB 1064, Nigeria; hbgaladima@unimaid.edu.ng; 9 Department of Veterinary Services, Ministry of Agriculture & Natural Resources, Kano PMB 3978, Nigeria; abunaima2013@gmail.com; 10 Department of Veterinary Public Health and Preventive Medicine, Faculty of Veterinary Medicine, University of Abuja, Abuja 900109, Nigeria; chinwe4greatness@gmail.com (C.E.O.); elishak555@gmail.com (E.Z.K.); 11 Department of Animal Production, Faculty of Agriculture, Delta State University of Science and Technology, Ozoro PMB 005, Delta State, Nigeria; onyiionwumere@yahoo.com; 12 Department of Medicine and Surgery, College of Medicine, University of Ibadan, Ibadan 200005, Nigeria; priscawoyomi023@gmail.com; 13 Department of Veterinary Public Health and Preventive Medicine, Faculty of Veterinary Medicine, Usmanu Danfodiyo University, Sokoto PMB 2346, Nigeria; aliyu.musawa@udusok.edu.ng (M.A.I.); mdsal70@gmail.com (M.D.S.); 14 Department of Veterinary Public Health and Preventive Medicine, Faculty of Veterinary Medicine, Ahmadu Bello University, Zaria PMB 1044, Nigeria; kaboskylawal@yahoo.com; 15 Department of Animal Health and Technology, Bauchi State College of Agriculture, Bauchi PMB 0088, Nigeria; sazailani2@gmail.com; 16 LYSSA LLC, Atlanta, GA 30044, USA; charleserupprechtii@gmail.com

**Keywords:** academics, *Coronavirus*, COVID-19, epidemiology, healthcare workers, Nigeria, SARS-CoV-2 infection, tertiary students, vaccine hesitancy

## Abstract

The COVID-19 pandemic has resulted in millions of human deaths, prompting the rapid development and regulatory approval of several vaccines. Although Nigeria implemented a COVID-19 vaccination program on 15 March 2021, low vaccine acceptance remains a major challenge. To provide insight on factors associated with COVID-19 vaccine hesitancy (VH), we conducted a national survey among healthcare workers, academics, and tertiary students, between 1 September 2021 and 31 December 2021. We fitted a logistic regression model to the data and examined factors associated with VH to support targeted health awareness campaigns to address public concerns and improve vaccination rates on par with global efforts. A total of 1525 respondents took part in the survey, composed of healthcare-workers (24.5%, 373/1525), academics (26.9%, 410/1525), and students (48.7%, 742/1525). Only 29% (446/1525) of the respondents were vaccinated at the time of this study. Of the 446 vaccinated respondents, 35.7% (159/446), 61.4% (274/446) and 2.9% (13/446) had one, two and three or more doses, respectively. Reasons for VH included: difficulty in the vaccination request/registration protocols (21.3%, 633/1079); bad feelings towards the vaccines due to negative social media reports/rumours (21.3%, 633/1079); personal ideology/religious beliefs against vaccination (16.7%, 495/1079); and poor confidence that preventive measures were enough to protect against COVID-19 (11%, 323/1079). Some health concerns that deterred unvaccinated respondents were: innate immunity issues (27.7%, 345/1079); allergic reaction concerns (24.6%, 307/1079); and blood clot problems in women (21.4%, 266/1079). In the multivariable model, location of respondents/geopolitical zones, level of education, testing for COVID-19, occupation/job description and religion were significantly associated with VH. Findings from this study underscore the need for targeted awareness creation to increase COVID-19 vaccination coverage in Nigeria and elsewhere. Besides professionals, similar studies are recommended in the general population to develop appropriate public health interventions to improve COVID-19 vaccine uptake.

## 1. Introduction

Globally, approximately 350 million people have been infected with SARS-CoV-2 and more than seven million have died [1]. Both infection and mortality rates are under-reported, especially in developing countries with low testing. Most reported infections and mortalities were recorded in the Americas, Europe, and Asia. Africa reported the least number of infections and deaths [1]. In Nigeria, over 254,000 confirmed cases and 3100 deaths were reported as of 14 March 2022 [2]. Most occurred in Lagos State, the Federal Capital Territory (FCT, Abuja) and Rivers State. Historically, mass vaccination was an effective strategy for disease prevention [3,4]. Mass vaccination campaigns were used to eliminate several diseases in Nigeria, such as polio. Typically, at least 70% of the population requires herd immunity to interrupt pathogen transmission [5]. Therefore, VH, considered a delay in acceptance or refusal of vaccination, despite the availability of such services, may disrupt the desired public health outcome for disease prevention, control, and eventual elimination.

The Nigerian government continued to advocate the implementation of non-pharmaceutical measures, including contact tracing, quarantine, and COVID-19 vaccination on 15 March 2021, to flatten the epidemic curve. Despite these efforts, waves of infection occurred, with a 4th wave driven by the omicron variant. As of February 2022, the national vaccination coverage was only 3%, extremely low compared to Canada, Chile, France, Italy, Japan, Portugal, Spain, and the U.K., with over 70% vaccination [1]. There are a few regional reports on COVID-19 VH, such as among staff at a tertiary institution in South East Nigeria and among HIV patients [6,7,8,9]. However, no national study was undertaken to understand factors associated with VH, as inferential for public health policy decisions.

Consequently, this study aimed to unravel factors associated with COVID-19 VH among Nigerian healthcare workers, academics, and tertiary students, and also determined their views and beliefs concerning vaccination. Assumedly, such data from these professionals are vital to support SARS-CoV-2 prevention strategies and may help guide policy formulation to overcome VH in Nigeria. 

## 2. Materials and Methods

### 2.1. Study Area

**Figure 1:**
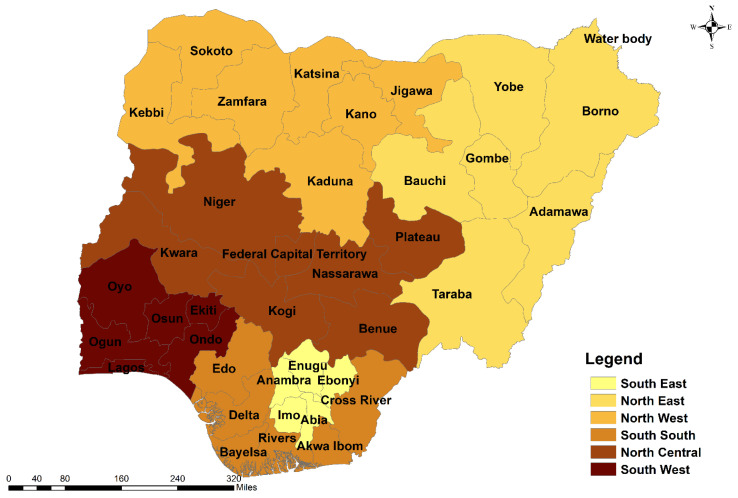
Map of Nigeria showing the six geopolitical zones, the constituent 36 states and the Federal Capital Territory (FCT), Abuja.

Nigeria is a West African nation on the Gulf of Guinea in sub-Saharan Africa. The country is located at Latitude 9°04′39.90″ N and Longitude 8°40′38.84″ E, and experiences a temperature range of 16 °C to 45 °C. Nigeria is made up of 36 States and the FCT, Abuja. The 36 states and the FCT are grouped into six regional or geopolitical zones: Southeast (SE); South-south (SS); Southwest (SW); Northeast (NE); Northwest (NW); and North-central (NC) (Figure 1).

### 2.2. Study Design, Study Population and Sample Size Determination

**Figure 2:**
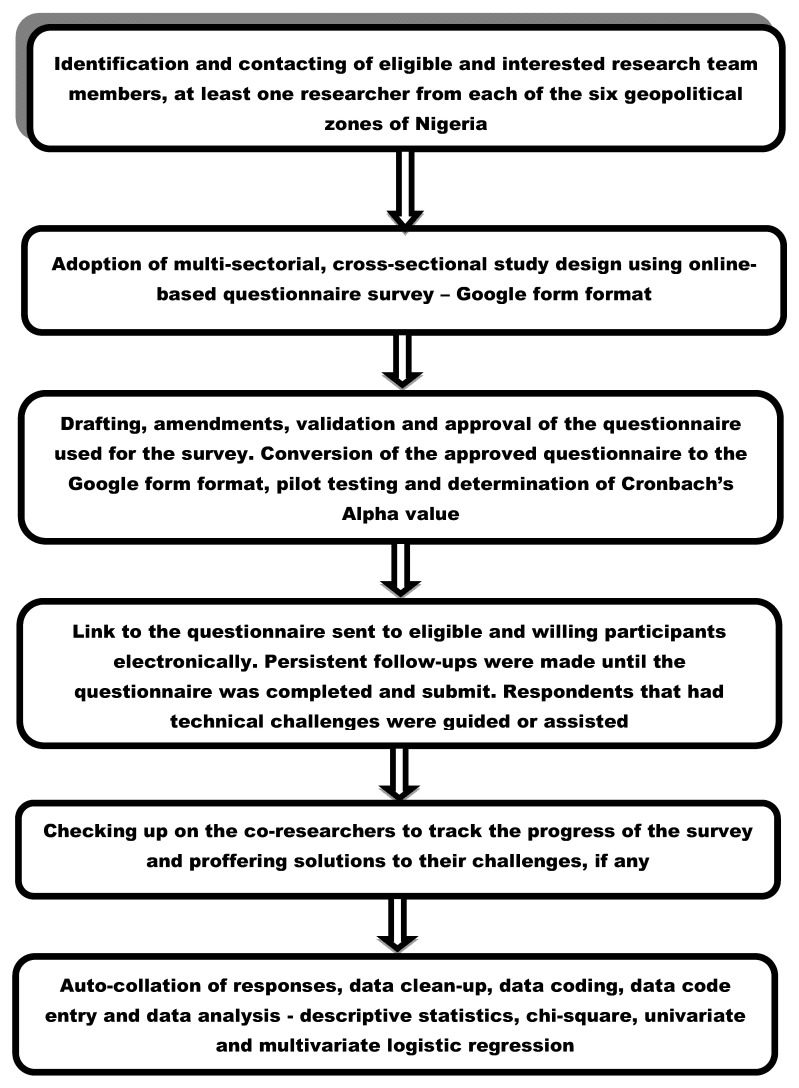
Schematic overview for the assembly of the research team, study design, and data acquisition procedures during the online-based questionnaire survey.

This study adopted a multi-sectorial, cross-sectional study design using an online-based questionnaire. The survey was conducted between September and December 2021. Healthcare workers (i.e., medical and veterinary doctors, nurses, pharmacists, and laboratory scientists/technologists), academics (i.e., teaching staff in tertiary institutions), and tertiary students (i.e., those enrolled in universities, polytechnics, and colleges of education), resident in any part of Nigeria, comprised the study population. A minimum sample size (MSS) of 423 was computed using an online software sample size calculator^®^ (https://www.surveysystem.com/sscalc.htm, accessed on 6 January 2022). Lacking national data on COVID-19 testing, infection and vaccine acceptance or VH among the selected study population, the MSS computation assumed 50% prevalence and was benchmarked on a 95% confidence interval and a 5% margin of error. Additionally, provision for 10% non-contingency was factored in during the MSS estimation. Although 423 was the MSS calculated, a total of 1525 respondents were surveyed for robustness and data accuracy. An overview of the study design and procedures are presented schematically in Figure 2.

### 2.3. Questionnaire Design, Validation, and Pilot Testing

A closed-ended Google form questionnaire (Alphabet Incorporated, Mountain View, CA, USA) was used as the instrument of collection. The form consisted of 27 questions written in English and categorized into five parts. The first part consisted of seven questions that focused on the socio-demographics of the respondent, including: gender; state of residence; job description; students’ years of study; age; marital status; religion; and highest educational status. The second and third parts focused on the COVID-19 infection status of the respondents (four questions) and availability and accessibility of COVID-19 vaccine in the respondents’ locality (six questions), respectively. Information on COVID-19 vaccine acceptance and VH (eight questions) and ways to improve COVID-19 availability and acceptance (one question) were the fourth and fifth parts of the questionnaire. A complete copy of the question is attached as supplementary material (Table S1). 

After the design, the questionnaire was subjected to face and content validations, following the method described by Bolarinwa [10]. A six-person panel of experts, conversant with the subject area and selected from different parts of the country, reviewed the questionnaire, scored each question based on relevance and clarity and made recommendations. From these scores, the scale-cumulative validity index (s-CVI) and mean item-cumulative validity index (mean i-CVI) were calculated, as described by Zamanzadeh et al. [11]. The computed s-CVI and mean i-CVI for relevance were 0.92 and 0.92, respectively (i.e., values greater than 0.9 indicated that the questionnaire contents were relevant and therefore required no modification). Similarly, the calculated s-CVI and mean i-CVI values for clarity were 0.81 and 0.88, respectively. Thereafter, some questions were revised as recommended to enhance clarity. Additionally, the questionnaire was pilot tested on 30 respondents prior to the survey, and errors were corrected. Afterwards, a Cronbach’s Alpha test was performed. This yielded an alpha-value of 0.72 (more than 0.6 benchmarks) and further confirmed the questionnaire validity in obtaining the parameters of interest. Similarly, the reliability/consistency of the questionnaire was determined by the test-retest method [12].

### 2.4. Sampling Procedure

This survey was conducted in compliance with the Checklist for Reporting Results of Internet E-Surveys and procedures for ethical practice for conduct and reporting of web-based studies [13]. The inclusion criteria were that respondents must be ≥16 years old and a Nigerian-based healthcare worker, academic staff member, or tertiary student. To prevent duplicate or multiple responses, the Google Form settings were adjusted to allow respondents having the same internet protocol address only one access to the questionnaire. To ensure complete responses, the setting was also adjusted such that successful submission was only possible when all questions have been answered. 

A link to the Google form questionnaire was sent to the eligible participants electronically (i.e., via email and social media). Bulk email systems of various universities, group WhatsApp, and direct electronic communication with eligible participants were the major means to reach respondents. Reminder emails and follow up chats/calls to respondents were made periodically, to track survey progress. Completion of the form was deemed easy and seamless, but logistical assistance was provided to a few respondents who drew the attention of the researchers to technical glitches in completing the form. A minimum of 1000 respondents, which consisted of 500 tertiary students and 250 academics and healthcare workers each, were targeted and invited to complete the questionnaire in each of the six geographical zones nationwide. However, acceptance of the invitation and completion of the questionnaire were solely at the discretion of the invitees.

### 2.5. Ethical and Informed Consent Considerations

Human subjects ethical approval did not apply to this web-based survey, as the researchers did not see or handle respondents directly. Prior to the survey, informed consent to participate in the survey was sought on the first page of the questionnaire. Respondents who agreed to partake in the study were urged to complete and submit the questionnaire. Participation in the study was entirely voluntary and at the respondents’ discretion. There was no penalty for non-participation, and contact information, or personal identities of the respondents (i.e., email address, phone numbers) were not collected. The study complied with the World Medical Association Declaration of Helsinki of 2013 [14].

### 2.6. Data Analyses

Responses from the completed and submitted Google Form were automatically collated on an Excel spread sheet. After data clean-up, the responses were coded for the descriptive and inferential statistical analyses. Descriptive statistics (frequencies and percentages) were used to compute the socio-demographic data (i.e., gender, job description, age, marital status, religion, and educational level) and the results were presented in tables, bar charts, and pie charts. To provide insights into association between COVID-19 vaccine hesitancy and variety independent variables, we categorized the response ‘are you willing to take COVID-19 vaccine’ as our outcome of interest (mean 0.58, SE, 0.12 95%Cl, 0.56–0.61) and constructed a univariable logistic regression model using the open-source software R (version 3.6. 2) in the RStudio environment. All significant variables based on a conservative p-value (i.e., 0.20) in the univariable analysis were considered for inclusion in the multivariable model. We fitted the multivariable model using a backwards stepwise approach to create a main effects model using a significance level of 0.1 and retained confounders regardless of statistical significance. We assessed confounding by examining the change in the coefficients for the remaining significant variables after removing the potentially confounding variable. If the coefficient for one of these variables changed more than 20%, the removed variable was considered a confounder and was retained in the model. A value of < 0.05 was deemed significant. We assessed the model fit after completing the final multivariable model using the Akaike Information Criterion (AIC).

## 3. Results

### 3.1. Percentage Response Rate

The 6000 eligible respondents were targeted nationwide by using the questionnaire link via bulk email and WhatsApp groups. However, an overall response rate of 25.4% (1525/6000) was recorded after the researchers shared the link directly to eligible participants and sent weekly follow up reminder notices.

### 3.2. Socio-Demographics

**Figure 3:**
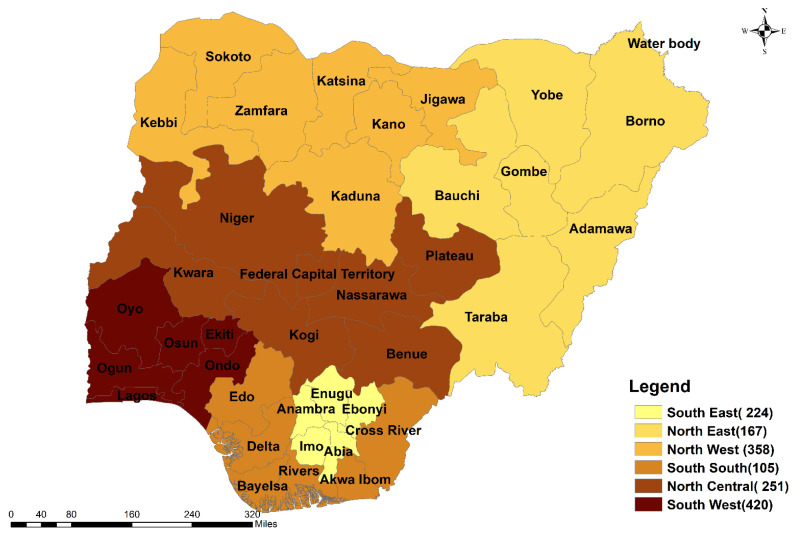
Distribution of the number of respondents across the six geopolitical zones of Nigeria.

**Figure 4:**
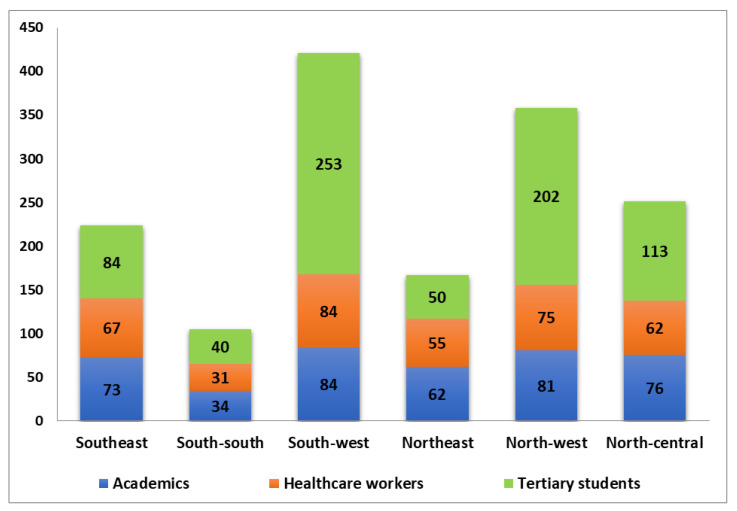
Distribution of the number of respondents (academics, healthcare works and tertiary students) surveyed for COVID-19 vaccine hesitancy across the six geopolitical zones of Nigeria.

A total of 1525 respondents, mostly male (1031, 67.6%) participated in the study. The distribution of the total number of respondents across the six geopolitical zones is shown in Figure 3. Respondents’ occupations/job descriptions and the corresponding proportions were: healthcare workers (24.5%, 373/1525); academics (26.9%, 410/1525); and students (48.7%, 742/1525). The distribution of the number of respondents for each of the three study populations (academics, healthcare works and tertiary students) across the six geopolitical zones is presented in Figure 4. The majority (52%, 793/1525) of the respondents were aged 16–30 years (Table 1). Most (35.7%, 545/1525) respondents were postgraduates, of the Christian faith (59.3%, 905/1525) (Table 1). There were significant associations (*p* < 0.05) between testing for SARS-CoV-2 infection and socio-demographics (Table 1). Similarly, there were significant positive associations (*p* < 0.05) among COVID-19 vaccination and occupation, age category, educational level, and geographical location (Table 1). 

### 3.3. SARS-CoV-2 Testing and Infection Status of Respondents

**Figure 5:**
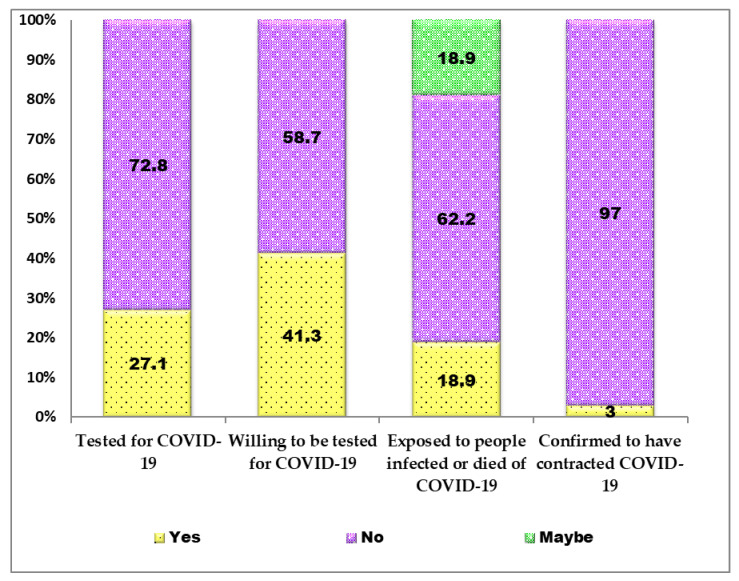
SARS-CoV-2 testing and infection status of Nigerian healthcare workers, academics and tertiary students (*n* = 1525) surveyed for COVID-19 vaccine acceptance and hesitancy.

Only 27.1% (401/1525) of the respondents had tested positive (i.e., by PCR) for SARS-CoV-2 infection (Figure 5). Other details, as well as information on exposure of the respondents to people confirmed to have contracted COVID-19 or died of the disease, are shown in Figure 5. 

### 3.4. COIVD-19 Vaccine Availability, Accessibility, Acceptance, and VH

**Figure 6:**
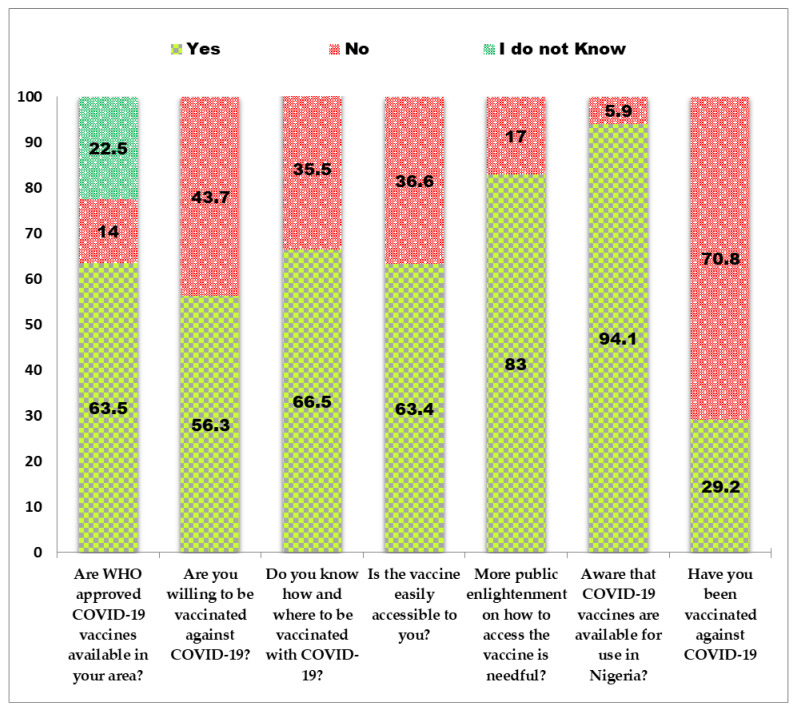
COIVD-19 vaccine availability, accessibility, acceptance, and hesitancy among healthcare workers, academics, and tertiary students in Nigeria.

**Figure 7:**
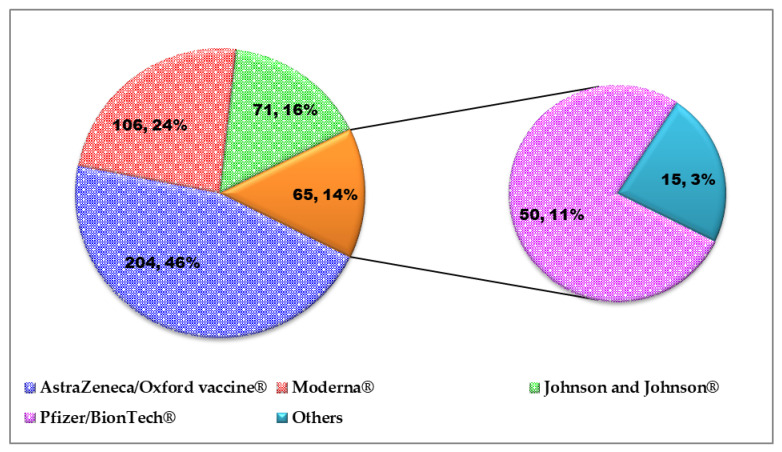
Distribution of COVID-19 vaccine brands received by vaccinated respondents (*n* = 446) surveyed for vaccine hesitancy in Nigeria.

**Figure 8:**
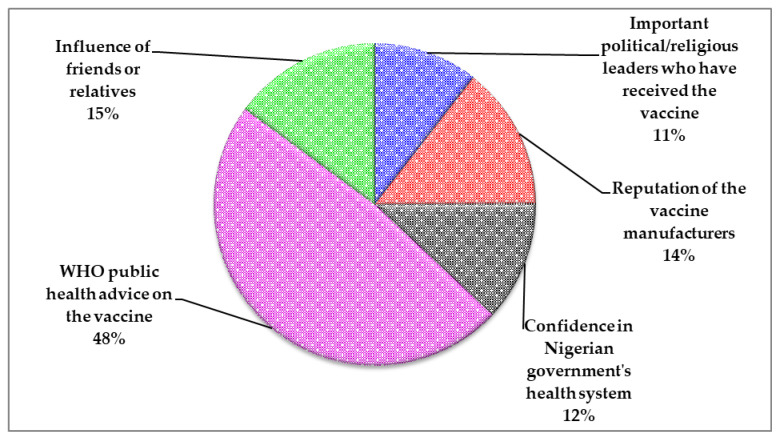
Reasons that informed the confidence of COVID-19 vaccinated respondents on the safety and efficacy of the vaccine.

Most (63.5%, 968/1525) respondents reported that WHO-approved COVID-19 vaccines were available for use in their locality (Figure 6). Only 29.2% (446/1525) of those surveyed were vaccinated at the time of their response. Of the 446 vaccinated respondents, 35.7% (159/446), 61.4% (274/446) and 2.9% (13/446) of respondents had received one, two and three or more inoculations, respectively. Details on vaccine brands received and the proportion of the recipients are presented in Figure 7. The major reason that informed the confidence of COVID-19 vaccinated respondents on the safety and efficiency of the vaccine was the WHO public health advice on the vaccine (48%) (Figure 8).

### 3.5. COVID-19 VH and Measures to Improve Its Availability and Acceptance in Nigeria

Comprehensive results on COVID-19 VH and ways to improve the availability and acceptance in Nigeria are presented in Table 2. Table 3 contains the results on univariable and multivariate analyses. The multivariable model indicated a spatial bias, with 2.4 odds of VH for respondents in the north-eastern part of Nigeria (AOR, 2.44, 95% CI, 1.53–3.92, *p* ≤ 0.001) versus those from the southwest. Compared to health professionals, academics had 1.7 odds of VH (AOR, 1.73, 95% CI, 1.19–2.53, *p* = 0.011). The odds of VH was 2.0 among undergraduate students (AOR, 2.02, 95% Cl, 1.42–2.90, *p* ≤ 0.001), compared to postgraduates. The geographical location, religion, occupation, level of education, and previous exposure of respondents were strongly associated with VH.

## 4. Discussion

This work represents a nationwide report on SARS-CoV-2 infection rates, COVID-19 VH, and SARS-CoV-2 infection among healthcare-workers, academics, and tertiary students in Nigeria. The geographical location, religion, occupation/job description, level of education and previous exposure of respondents were strongly associated with COVID-19 VH.

Our survey indicated that only 29% of the respondents were vaccinated against COVID-19. This finding is higher than previous reports from other African countries. For example, Afolabi et al. [15] reported that only 0.3% of the total population of the 15 countries in the Economic Community of West African States (ECOWAS) were fully vaccinated, three months after the commencement of COVID-19 in the region. If all ECOWAS countries followed this trajectory, the sub-region would have less than 1.6% of the total population fully vaccinated after 18 months of vaccination drives [15]. Of concern was the low vaccination rates among academics and healthcare professionals who were expected to drive the vaccination advocacy and enlightenment campaigns as primary stakeholders. Unexpectedly, in our multivariable model, academics were 1.7 times more likely to show VH towards COVID-19 vaccines compared to healthcare workers. Healthcare workers were assumed to be at a greater risk of COVID-19 than academics. Moreover, academics are likely to have somewhat different access to diverse resources, such as those which may perpetuate the ‘infodemic’, flooded with unsubstantiated conspiracy theory materials [16]. In our model, individuals who tested for COVID-19 were less likely to be reluctant to take the vaccine. Previous studies have shown that people who consider a disease terrifying are more likely to demand a vaccine against the disease [17]. Creating public awareness using people with previous disease experience may increase vaccination uptake. 

Our results indicate that respondents from the Northeast were 2.4 times more likely to be reluctant to receive COVID-19 vaccine. This finding may be attributed to the relatively low level of education in the Northern part of Nigeria. Previous disease control that relied on vaccination, such as polio, received significant setbacks in this region, which delayed control efforts [18]. To attain optimal vaccination coverage in Northern Nigeria, there is a need to engage religious and traditional leaders, who played a crucial role in polio elimination [19].

In this study, undergraduate students were more likely to hold VH opinions. Such undergraduates often rely solely on their lecturer for information than postgraduate students, who may be expected to hold a more independent view. In addition, undergraduate students are also influenced by their lecturers and their religious leaders [20]. Hence, the 29% vaccination rate was worrisome among academics and healthcare workers, who may shape the opinion of their subjects and hence local society in general due to the nature of their professions. Therefore, there is a need for more evidence-based enlightenment campaigns to these professionals on the safety and efficacy of COVID-19 vaccines. Moreover, social media reports that may be negatively influencing the perception of the respondents on COVID-19 vaccination need to be more balanced to improve vaccination rates [21].

Clearly, VH is a complex global public health problem that varies across cultures, time, places, and types of vaccines. Still, it is generally influenced by factors like complacency, convenience, and over confidence [22]. In retrospect, VH may be responsible for lower COVID-19 vaccination rates. The VH may not just be among the surveyed groups, but among the Nigerian population generally. As of 22 January 2022, data hosted on the WHO COVID-19 dashboard [1] indicated that the total vaccine doses administered and persons fully vaccinated per 100 population in Nigeria, were only nine and three, respectively. This number of persons fully vaccinated per 100 population is extremely low compared to the global average of 52 persons [1]. In Chile, the Republic of Korea, Portugal, Malaysia, Argentina and South Africa, the number of persons fully vaccinated per 100 population, as of 22 January 2022, were 87, 84, 83, 78, 73 and 27, respectively. From these figures, Nigeria is seriously behind regarding COVID-19 vaccination rates and VH may be responsible. The unwillingness of 44% of the unvaccinated respondents to be vaccinated, despite knowing how and where to be vaccinated, confirmed that COVID-19 VH exists in Nigeria.

Apart from VH, the limited number of vaccines available for use in the country in relation to the nation’s population and difficulty in accessing the vaccines may be contributory to the low vaccination rate found in this study. Due to the increased global demand for COVID-19 vaccine, African and other developing countries have been experiencing acute vaccine shortages as vaccine producers have struggled to cope with the increased demands and competition with wealthier countries due to upfront payments/orders made by these nations [23]. Nigeria has largely depended on vaccine donations from developed countries and the COVID-19 Vaccines Global Access (COVAX) facility. Although these donations have been helpful, it is challenging to meet the COVID-19 vaccine needs with an estimated population of 215 million [24]. Donated vaccines may have a limited shelf life, such that many expire before they are administered [25]. This underscores a need for indigenous vaccine production facilities to meet the local demand for COVID-19 and several other vaccines. 

Our results also indicated that religion was strongly associated with VH. Misconceptions and personal/religious beliefs of the respondents against the COVID-19 vaccine may have also contributed to the apathy or low vaccination rates recorded. Such misconceptions and the resultant low vaccination rate found in this survey may not be unconnected with adverse social media reports and unfounded rumours being circulated against COVID-19 vaccines [26]. Negative media may dampen interest in vaccines and result in rejection. Moreover, use of mRNA technology in vaccine production was relatively new, and concerns on the health or long-term immunological effects of these biologics have been a subject of controversy and scientific debate [27,28,29,30]. Reports published in 2021 showed that some healthcare workers from the USA and France were opposed to COVID-19 vaccination due to safety concerns associated with their rapid production and rollout [13,31]. 

Comparatively, COVID-19 VH was perceived as a major health problem in high or middle-income countries [32], while developing nations were being confronted with lack of access to the vaccines [33]. However, our findings revealed that Nigeria is challenged with both COVID-19 VH and limited vaccine access, as about 36% of the respondents did not know how and where to get vaccinated. The synergy of these two hurdles may negatively influence the COVID-19 trajectory by worsening community transmission. Community transmission of SARS-CoV-2, referring to the inability to relate large numbers of confirmed cases to any known chain of viral transmission, is a significant problem that may enhance the emergence of new strains/variants [34]. To buttress the importance of COVID-19 vaccination, Singanayagam et al. [34] reported significant reduction in community transmission of the alpha variant (B.1.1.7) by up to 50% in infected and vaccinated people. The reduction in SARS-CoV-2 viral load in infected and the vaccinated group than in the infected and unvaccinated group suggested that vaccination may have lowered viral infectiousness [34]. Persistent pools of unvaccinated populations globally may constitute a potential risk for emerging and disseminating new variants of concern, such as Omicron. For example, in South Africa, where the Omicron variant was first reported in November 2021 [35], the number of COVID-19 vaccine doses administered weekly reduced to less than one-quarter of the number given earlier during the peak of the vaccination drive [36] As such, COVID-19 VH in Nigeria may be perpetuating community transmission of SARS-CoV-2.

Although emergency use and repurposing of certain drugs for management of COVID-19 have been granted [37,38,39], mass vaccination remains the best and the most cost-effective means of controlling infectious diseases, especially with pandemic status [40,41]. This underscores the need for grass-roots public education, beginning with healthcare workers, academics, and tertiary students. Public education campaigns should be led by an independent body, with international standing, and be based solely on the scientific evidence regarding the safety and efficacy of COVID-19 vaccines. This should help clarify vaccine misconceptions and enhance acceptance in Nigeria and elsewhere, for broader vaccination coverage towards the attainment of herd immunity. 

Moreover, COVID-19 vaccines should be seen as a “global public/common good” which should be available and accessible to everyone, notwithstanding their national or personal economic status [42]. Although vaccine manufacturers deserve income, bottlenecks and challenges that constrain the availability and accessibility of vaccines, especially in developing countries, should be removed. This includes vaccine nationalization and national/local or global politics that may have limited COVID-19 vaccine availability. Liberalization of access to vaccination is critically important because the infected, unvaccinated populations may constitute reservoirs or “mixing vessels” for viral reassortment, which may enhance the emergence and dissemination of new variants, further endangering global health [43]. While the topic of VH is of obvious relevance to the current pandemic, the issue is applicable to vaccines in general [40], whether related to disease eradication at a population level, as with polio regionally [19], or on an individual basis, such as the recent refusal of prophylaxis by a person bitten by a rabid animal in the USA [43]. 

Our findings should be interpreted within the context of the limitations of a questionnaire-based study, including respondents with biases. Results from such online surveys cannot be generalized to the population because respondents may not be truthful with their responses. Additionally, there are individual challenges associated with understanding and interpretation of the questions. As a single cross-sectional study, we cannot predict how VH will change over time.

## 5. Conclusions

This study provided insight into factors associated with COVID-19 VH among healthcare workers, academics, and tertiary students. The geographical location, religion, occupation, level of education, and previous exposure of respondents were strongly associated with VH. Findings from this study underscore the need for targeted awareness creation to increase COVID-19 vaccination coverage in Nigeria. Similar studies in the general population are recommended to develop appropriate public health interventions to improve COVID-19 vaccine uptake.

## Figures and Tables

**Table 1:** Association between socio-demographics of respondents (*n* = 1525) and testing for SARS-CoV-2 infections and COVID-19 vaccination.

Socio-Demographics	Variables	Proportion	χ^2^-Value	*p*-Value
EVER TESTED FOR SARS-CoV-2				
**Gender**	Male	67.6% (1031/1525)	0.371	0.543
	Female	32.4% (494/1525)		
**Job description**	Healthcare worker	24.5% (373/1525)	16.82	0.002 *
	Academic	26.9% (410/1525)		
	Tertiary student	48.7% (742/1525)		
**Age categories (years)**	16–30	52% (793/1525)	149.1	<0.001 *
	31–45	34.3% (523/1525)		
	≥46	13.7% (209/1525)		
**Marital status**	Single	52.4% (799/1525)	7.347	0.007 *
	Married/divorced	47.6% (726/1525)		
**Religion**	Christianity	59.3% (905/1525)	1.484	0.223
	Islam/others	40.7% (620/1525)		
**Educational level**	Postgraduate	35.7% (545/1525)	13.27	0.001 *
	Graduate	29.1% (443/1525)		
	Undergraduate	35.2% (537/1525)		
**Location**	South-west	6.9% (105/1525)	19.46	0.0006 *
	South-south	14.7% (224/1525)		
	Southeast	11.0% (167/1525)		
	Northeast	23.5% (358/1525)		
	North-west	16.5% (251/1525)		
	North-central	6.9% (105/1525)		
**VACCINATION AGAINST COVID-19**				
**Gender**	Male	67.6% (1031/1525)	1.299	0.254
	Female	32.4% (494/1525)		
**Job description**	Healthcare worker	24.5% (373/1525)	34.43	0.000 *
	Academic	26.9% (410/1525)		
	Tertiary student	48.7% (742/1525)		
**Age categories (years)**	16–30	52% (793/1525)	151.4	<0.001 *
	31–45	34.3% (523/1525)		
	≥46	13.7% (209/1525)		
**Marital status**	Single	52.4% (799/1525)	0.881	0.348
	Married/divorced	47.6% (726/1525)		
**Religion**	Christianity	59.3% (905/1525)	2.337	0.126
	Islam/others	40.7% (620/1525)		
**Educational level**	Postgraduate	35.7% (545/1525)	16.17	0.003 *
	Graduate	29.1% (443/1525)		
	Undergraduate	35.2% (537/1525)		
**Location**	South-west	6.9% (105/1525)	24.94	<0.001 *
	South-south	14.7% (224/1525)		
	Southeast	11.0% (167/1525)		
	Northeast	23.5% (358/1525)		
	North-west	16.5% (251/1525)		
	North-central	6.9% (105/1525)		

* Statistically significance *p*-value; Chi-square test.

**Table 2:** Reasons for COVID-19 vaccine hesitancy and measures to improve the vaccine availability and acceptance in Nigeria.

S/No	Questions Asked or Information Required	Number of Respondents (%)
**1.**	* Reasons for non-vaccination among unvaccinated respondents (*n* = 1079)	
	COVID-19 vaccination registration protocol is difficult	633 (58.7)
	Suspicion/doubts on safety of novel vaccines	43 (4.0)
	COVID-19 is a hoax	183 (17.0)
	The vaccines are not available/accessible in my locality	57 (5.3)
	Herbal medicines/home remedies are effective for cure/management of COVID-19	65 (6.0)
	Influence from anti-COVID-19-vaccine movements	40 (3.7)
	Vaccination is against my religious beliefs or personal ideology	495 (45.9)
	Concerns about long term health/side effects	296 (27.4)
	Scepticism about the vaccine due to hasty production/roll out	159 (14.7)
	Preventive measures are enough to protect against COVID-19	323 (30.0)
	Bad feelings towards the vaccines due to negative social media reports/rumours	633 (58.7)
	Others	37 (3.4)
**2.**	* Some health concerns that prevented unvaccinated respondents (*n* = 1079) from getting vaccinated	
	Blood clot issues among women	266 (24.7)
	Allergic reactions	307 (28.5)
	Innate immunity concerns	345 (32.0)
	New or worsening muscle/joint pains	107 (10.0)
	Myocardial infarction	180 (16.7)
	Others	41 (3.8)
**3.**	* Fears against COVID-19 vaccine among unvaccinated respondents (*n* = 1079)	
	The vaccines may be unsafe due to its hasty production and or roll out	674 (62.9)
	The vaccine may contain ‘hidden chip’ that may be a mark of the anti-Christ	208 (19.3)
	It may limiting procreation and fertility and hence a population control strategy	206 (19.9)
	The vaccine may cause deaths as one certain vaccine did in Kano State, Nigeria	132 (12.2)
	The vaccine may cause adverse immunological problem as mRNA vaccine are relatively new and sufficient time is needed to proof their safety and efficacy	613 (56.8)
	Others	22 (2.0)
**4.**	* Ways to improve COVID-19 vaccine availability and acceptance in Nigeria (*n* = 1525)	
	More COVID-19 vaccine donations from developed to developing countries	519 (34.0)
	Increased funding to the Nigerian health and education/research sectors	727(47.7)
	More public enlightenment campaigns/grass-root health education to the populace	955 (62.6)
	Procurement of more COVID-19 vaccines	379 (24.8)
	Commencement of indigenous COVID-19 vaccine production	505 (33.1)
	Provision of COVID-19 vaccine storage and distribution facilities	453 (29.7)
	Increased remuneration and provision of incentives to healthcare workers	437 (28.7)
	Developed countries should halt 3rd COVID-19 booster vaccination to increase the availability of the vaccines in developing countries	211 (13.8)

* Respondents were allowed to provide more than one response, as may be appropriate.

**Table 3:** Univariable and multivariable results of variables associated with COVID-19 vaccine hesitancy among Nigerian healthcare workers, academics, and tertiary students.

Univariable Analysis					Multivariable Model	
Variables	Categories	Proportions	OR (95% CI)	*p*-Value	AOR (95% CI)	*p*-Value
**Gender**	Male	67.6% (1031/1525)	Referent	Referent	Referent	Referent
	Female	32.4% (494/1525)	1.28 (1.03–1.60)	0.026	1.13 (0.88–1.45)	0.30
**State**	South West	27.5% (422/1525)	Referent	Referent	Referent	Referent
	South-South	6.9% (105/1525)	1.03 (0.67–1.61)	0.90	1.12 (0.71–1.79)	0.60
	South East	14.7% (224/1525)	1.42 (1.01–2.01)	0.044	1.60 (1.11–2.32)	0.012
	North East	11.0% (167/1525)	1.24 (0.86–1.81)	0.30	2.40 (1.51–3.87)	<0.001
	North West	23.5% (358/1525)	0.61 (0.46–0.81)	<0.001	1.31 (0.88–1.95)	0.20
	North Central	16.5% (251/1525)	0.89 (0.65–1.22)	0.50	1.28 (0.91–1.80)	0.20
**Job description**	Healthcare worker	24.5% (373/1525)	Referent	Referent	Referent	Referent
	Academic	26.9% (410/1525)	1.05 (0.82–1.35)	0.70	1.19 (0.85–1.67	0.3
	Tertiary student	48.7% (742/1525)	0.86 (0.67–1.10)	0.20	1.73 (1.13–2.65)	0.012
**Age categories (years)**	16–30	52.0% (793/1525)	Referent	Referent	Referent	Referent
	31–45	34.3% (523/1525)	0.68 (0.55–0.86)	<0.001	0.75 (0.51–1.10)	0.15
	≥46	13.7 (209/1525)	0.64 (0.47–0.88)	0.005	0.63 (0.37–1.05)	0.078
**Marital status**	Single	52.4% (799/1525)	Referent	Referent	Referent	Referent
	Married/divorced	47.6% (726/1525)	0.76 (0.62–0.93)	0.009	1.13 (0.78–1.62)	0.50
**Religion**	Christian	59.3% (905/1525)	Referent	Referent	Referent	Referent
	Non-Christian	40.7% (602/1525)	0.56 (0.45–0.69)	<0.001		
Highest educational level attained	Postgraduate	35.7% (545/1525)	Referent	Referent	Referent	Referent
	Graduate	29.0% (443/1525)	1.05 (0.81–1.35)	0.70	1.19 (0.85–1.67)	0.3
	Undergraduate	35.0% (537/1525)	1.51 (1.19–1.94)	<0.001	1.73 (1.13–2.65)	0.012
Tested against COVID-19	Yes (1)	26.3% (401/1525)	Referent	Referent	Referent	Referent
	No (0)	73.7% (1124/1525)	2.43 (1.90–3.13)	<0.001		

OR = Odds Ratio, AOR = Adjusted Odds Ratio, CI = Confidence Interval.

## Data Availability

The data supporting the findings of this study are available on reasonable request from the corresponding author, E.O.N.

## Supplementary Materials

The following supporting information can be downloaded at: https://www.mdpi.com/article/10.3390/vaccines10040626/s1, Table S1: Questionnaire.
