# Supplementary material for: COVID-19 Vaccine Hesitancy and Determinants of Acceptance among Healthcare Workers, Academics and Tertiary Students in Nigeria
Source: Vaccines (Basel). 2022 Apr 15;10(4):626. doi: 10.3390/vaccines10040626 (PMC9032510; doi:10.3390/vaccines10040626)
Supplement: Supplementary file 1 [file vaccines-10-00626-s001.zip › vaccines-1616821-supplementary.pdf]

COVID-19 Vaccine Acceptance and Hesitancy among Nigerian Healthcare Workers, Academics and Tertiary Students

Table S1. Questionnaire.

This survey is only for research purposes and the confidentiality of all respondents is fully guaranteed. So, be free and very sincere as you provide your responses to all the 27 questions

Please provide your response to the questions by ticking the appropriate option(s) in the response column

S/no	Questions or information required	Respons e
SOCIODEMOGRAPHICS		
1.	**Gender**	
	Male	
	Female	
2.	**State of residence in Nigeria**	
3.	**Occupation/Job description**	
	Healthcare worker	
	Academic	
	Student (higher/tertiary institutions)	
4.	**If you are a student, please specify your year of study**	
	< 3rd year (undergraduate)	
	≥ 3rd year (undergraduate)	
	Postgraduate	
	Not applicable	
5.	**Age category**	
	16-30	
	31-45	
	46-60	
	≥61	
6.	**Marital status**	
	Single	
	Married	
	Divorced	
7.	**Religion**	
	Christianity	
	Islam	
	Others	
8.	**Highest educational level attained**	

	Postgraduate	
	Graduate	
	Undergraduate	
COVID-19 TESTING AND INFECTION STATUSES		
**9.**	**Have you been tested for COVID-19?**	
	Yes	
	No	
**10.**	**Have you been confirmed to have contracted COVID-19 before?**	
	Yes	
	No	
**11.**	**If not tested yet, are you willing to be tested for COVID-19?**	
	Yes	
	No	
	Not applicable	
**12.**	**Were you exposed to people confirmed to have contracted COVID-19 or died of the disease?**	
	Yes	
	No	
	May be	
AVAILABILITY AND ACCESSIBILITY OF COVID-19 VACCINE		
**13.**	**Are WHO approved COVID-19 vaccine available in your area?**	
	Yes	
	No	
	I do not know	
**14.**	**Are you willing to be vaccinated against COVID-19?**	
	Yes	
	No	
**15.**	**Do you know HOW and WHERE to be vaccinated with COVID-19?**	
	Yes	
	No	
**16.**	**Is the vaccine easily accessible to you?**	
	Yes	
	No	
**17.**	***If the vaccine is not easily accessible to you, what could be the major cause(s)?**	
	Very limited supply of the vaccine	
	Do not know how and or where to access the vaccine	

	My age category is not among the priority group	
	Not applicable	
	Do not know how and where to register and access the vaccine	
18.	**Do you think that more public enlightenment and grass-root awareness on HOW and WHERE to access the vaccine in Nigeria is needful?**	
	Yes	
	No	
**COVID-19 VACCINE ACCEPTANCE AND HESITANCE**		
19.	**Are you aware that COVID-19 vaccines are available for use in Nigeria?**	
	Yes	
	No	
20.	**Have you been vaccinated against COVID-19**	
	Yes	
	No	
21.	**If vaccinated, which of the approved vaccines did you receive?**	
	AstraZeneca/Oxford vaccine®	
	Johnson and Johnson®	
	Moderna®	
	Pfizer/BionTech®	
	Sinopharm®	
	Sinovac®	
	Sputnik-V®	
22.	**If vaccinated, how many doses of the vaccine have you received?**	
	One	
	Two	
	More than two	
	Not applicable	
23.	***If vaccinated, what informed your confidence on the safety/efficiency of the vaccine?**	
	WHO public health advice on the vaccine	
	Important political/community/religious leaders who have received the vaccine	
	Reputation of the vaccine manufacturers	
	Confidence in Nigerian government/health system	
	Influence of friends or relatives	
	Not applicable	
24.	***If you are not vaccinated yet, why?**	
	COVID-19 vaccination request/registration protocol is difficult due to	

	limited internet access in some parts of the country	
	Suspicion/doubts on safety of novel vaccines	
	COVID-19 is a hoax	
	The vaccines are not available/accessible in my locality	
	Herbal medicines/home remedies are effective for cure/management of COVID-19	
	Influence from anti-COVID-19-vaccine movements	
	Vaccination is against my religious beliefs or personal ideology	
	Concerns about long term health/side effects	
	Scepticism about the vaccine due to hasty production/roll out	
	Preventive measures are enough to protect against COVID-19	
	Bad feelings towards the vaccines due to negative social media reports/rumours	
	Not applicable	
**25.**	***Some health concerns preventing me from getting vaccinated are:**	
	Blood clot issues among women	
	Allergic reactions	
	Innate immunity concerns	
	New or worsening muscle/joint pains	
	Myocardial infarction	
	People say the vaccine is not good	
	Not applicable	
**26.**	***Some of my fears against COVID-19 vaccine are:**	
	The vaccines may be unsafe due to its hasty production and or roll out	
	The vaccine may contain 'hidden chip' that may be a mark of the anti-Christ	
	It may limiting procreation and fertility and hence a population control strategy	
	The vaccine may cause deaths as one certain vaccine did in Kano State, Nigeria	
	Not applicable	
	The vaccine may cause adverse immunological problem as mRNA vaccine are relatively new and sufficient time is needed to proof their safety and efficacy	
**IMPROVEMENT OF COVID-19 AVAILABILITY AND ACCEPTANCE**		
**27.**	***In which ways can COVID-19 vaccine availability and acceptance be improved in Nigeria?**	
	More COVID-19 vaccine donations from developed to developing countries	

	Increased funding to the Nigerian health and education/research sectors	
	More public enlightenment campaigns/grass-root health education to the populace	
	Procurement of more COVID-19 vaccines	
	Commencement of indigenous COVID-19 production	
	Provision of COVID-19 storage and distribution facilities	
	Increased remuneration and provision of incentives to healthcare workers	
	Developed countries should halt 3rd COVID-19 booster vaccination to increase the availability of the vaccines in developing countries	
	Others (please specify)	

Respondents may provide more than one response as may be appropriate
